# Four alpha ganglion cell types in mouse retina: Function, structure, and molecular signatures

**DOI:** 10.1371/journal.pone.0180091

**Published:** 2017-07-28

**Authors:** Brenna Krieger, Mu Qiao, David L. Rousso, Joshua R. Sanes, Markus Meister

**Affiliations:** 1 Harvard Biophysics Program, Harvard Medical School, Boston, Massachusetts, United States of America; 2 Center for Brain Science and Department of Molecular and Cellular Biology, Harvard University, Cambridge, Massachusetts, United States of America; 3 Division of Biology and Biological Engineering, California Institute of Technology, Pasadena, California, United States of America; Dalhousie University, CANADA

## Abstract

The retina communicates with the brain using ≥30 parallel channels, each carried by axons of distinct types of retinal ganglion cells. In every mammalian retina one finds so-called "alpha" ganglion cells (αRGCs), identified by their large cell bodies, stout axons, wide and mono-stratified dendritic fields, and high levels of neurofilament protein. In the mouse, three αRGC types have been described based on responses to light steps: On-sustained, Off-sustained, and Off-transient. Here we employed a transgenic mouse line that labels αRGCs in the live retina, allowing systematic targeted recordings. We characterize the three known types and identify a fourth, with On-transient responses. All four αRGC types share basic aspects of visual signaling, including a large receptive field center, a weak antagonistic surround, and absence of any direction selectivity. They also share a distinctive waveform of the action potential, faster than that of other RGC types. Morphologically, they differ in the level of dendritic stratification within the IPL, which accounts for their response properties. Molecularly, each type has a distinct signature. A comparison across mammals suggests a common theme, in which four large-bodied ganglion cell types split the visual signal into four channels arranged symmetrically with respect to polarity and kinetics.

## Introduction

The retina communicates visual information to the brain through the action potentials of retinal ganglion cells (RGCs). This population of neurons consists of more than thirty distinct types, each of which covers the retina to reliably encode its part of the visual message [1,2]. Among the best recognized are the so-called alpha ganglion cells (αRGCs). Although their physiological characteristics vary from species to species (reviewed in [3]), they are recognizable as a distinct morphological class by their large cell bodies, stout dendrites and axons, large mono-stratified dendritic arbors, and high levels of neurofilament proteins [4,5]. Alpha RGCs also share certain physiological properties, such as a short response latency and fast conducting axons [6–8]. Thus the αRGCs are among the first to signal a new stimulus to the brain. Furthermore, the visual response of αRGCs involves a pronounced nonlinearity prior to summation over the receptive field center [9,10], owing in large part to rectification at the bipolar cell synapse [11]. RGCs with alpha-like morphology have now been confirmed in the retinas of over 30 mammalian species including humans [5]. This striking evolutionary conservation suggests that they play an essential role in visual processing.

Despite the considerable attention focused on this class of ganglion cells, some basic uncertainties remain. One question regards the number of types in the αRGC class. Early work on cat retina described two structural types, distinct primarily by the level of dendritic stratification in the inner plexiform layer [4]. These were identified with two functional types, the On- and Off-brisk-transient cells, named for their rapid light response [7]. Further morphological analysis in the rabbit retina suggested there may actually be four αRGC types [12], but this was not confirmed molecularly (e.g., by neurofilament staining) or physiologically. In the mouse retina, the consensus in the literature describes three αRGC types. Their visual responses are Off-transient, Off-sustained, and On-sustained [13,14]. If correct, this would imply an odd asymmetry of functional coverage, with an Off-channel that reports both transient and sustained changes, and an On-channel reporting only sustained signals. We thought it important to reexamine this claim because the mouse has become an increasingly important model animal in visual neuroscience, including an entire brain institute dedicated to understanding its visual cortex. The mouse αRGCs project to both the superior colliculus and the core region of the visual thalamus, and thus form a major input to central circuits for image processing [15]. Thus, there is great value in understanding the visual signals carried by this population.

One obstacle to a fuller understanding of αRGCs has been the absence of a genetic handle on the entire population. This limits one’s ability to target them for physiological recordings or to manipulate them prospectively. Here we used a mouse line in which αRGCs express Cre recombinase, allowing us to label them with a fluorescent reporter [16]. We found that the marked neurons in this line include all the previously known αRGC types. By targeted recording we discovered a fourth type in this class with different visual responses. The four αRGC types share many structural and functional features, including a distinctive waveform of the action potential. Finally, we used morphological and mosaic criteria to provide evidence that each of the αRGC groups constitutes an authentic cell type. A comparison of structural and functional properties across all four types reveals a remarkable symmetry in neural coding by this important class of ganglion cells.

## Materials and methods

### Mice

Both male and female mice were used, aged at approximately P21. Animals were maintained on a 12:12 light:dark cycle and fed standard mouse chow ad libitum. The KCNG4-Cre, TYW3, TYW7 and Thy1-STOP-YFP transgenic mouse lines were generated in our laboratory, and have been characterized previously [16–19]. The CB2-GFP line [20,21] was obtained from the Mutant Mouse Resource Research Center (MMRRC; https://www.mmrrc.org). Cre-positive cells in KCNG4-Cre were visualized by crossing with one of three cre-dependent reporter lines: Thy1-STOP-YFP line 1, RC-stop-EGFP (Ai3 [22]), or RC-stop-channelrhodopsin2-tdtomato (Ai23 [23]); Ai3 and Ai23 were obtained from Jackson Laboratories. The expression pattern varied somewhat depending on reporter line; the Thy1-STOP-YFP line 1 was most restricted to the ganglion cell layer. Mice were maintained on a C57BL/6J background.

This study was carried out in strict accordance with the recommendations in the Guide for the Care and Use of Laboratory Animals of the National Institutes of Health. The protocol was approved by the Institutional Animal Care and Use Committees at Harvard University (protocol 92–19) and the California Institute of Technology (protocol 1652). All efforts were made to minimize suffering.

### Electrophysiology

Mice were dark adapted for at least 1 hour prior to euthanasia by cervical dislocation. The retina was isolated under infrared illumination into Ames medium oxygenated with 95% O_2_, 5% CO_2_ at room temperature. A ~2–3 mm hole was cut into nitrocellulose filter paper and the retina was mounted over this aperture with ganglion cells (RGCs) facing up and placed in a superfusion chamber heated to 34–36 degrees C (unless noted otherwise). A two-photon microscope was used to identify fluorescent RGCs for loose-patch recording. Electrodes (2–5 MOhm) filled with Ames medium were used to record action potentials with a Multiclamp 700B amplifier (Molecular Devices). Custom programs in IGOR (Wavemetrics Inc.) were used for spike thresholding and analysis.

### Stimulation

Light stimuli were created using the Psychophysics Toolbox extensions in Matlab. A modified Texas Instruments Lightcrafter with a custom lens system focused the stimuli onto the photoreceptors (frame rate 60 Hz, magnification 9.1 μm/pixel). The average stimulus intensity expressed in photoisomerizations per second for each of the three mouse photoreceptors corresponds to 5.7 × 10^3^ R*/s for the rod, 2.1 × 10^3^ P*/s for the M cone, and 4.8 × 10^3^ P*/s for the S cone.

### Histology

After electrophysiological experiments, some retinas were fixed in fresh 4% paraformaldehyde in PBS at 4 degrees C for 1 hour. After fixation, the retinas were washed and incubated at 4 degrees C with primary antibodies for 4–5 days. Secondary antibody incubation at room temperature for at least 2 hours preceded mounting on a glass slide with spacers, ganglion cell side up, with Prolong Gold (Invitrogen). Whole mount images were obtained on a LSM 710 inverted NLO microscope at 20X or 40X (Zeiss). The primary antibodies used were: anti-GFP (rabbit, Life Technologies; chick, Abcam); mouse anti-Nonphosphoneurofilament H (SMI-32, Covance); goat anti-Osteopontin (R&D Systems); rabbit anti-Parvalbumin, rabbit anti-Calbindin, and mouse anti-Calretinin (all from Swant); anti-vAChT (goat, Promega; guinea pig, Millipore); goat anti-ChAT (Millipore); mouse anti-Brn3a (Millipore); goat anti-Brn3 (raised against Brn3b, Santa Cruz Biotechnology) and mouse anti-Brn3c (Santa Cruz Biotechnology). Dylight405-, Alexa488-, Cy3- and Alexa647-conjugated secondary antibodies were obtained from Jackson Immunoresearch.

## Results

### The KCNG4-Cre mouse line labels four types of alpha retinal ganglion cells (αRGCs)

We made use of a mouse line in which the gene for Cre recombinase was inserted into the locus encoding a potassium channel modifier, kcng4 [17]. After crosses to a reporter line (see Methods) double-transgenic mice expressed fluorescent protein in subsets of retinal neurons. With all three reporters tested, nearly all of the labeled neurons in the ganglion cell layer were RGCs with large somata and stout dendrites [16]. These features are suggestive of αRGCs [24]. In addition, these RGCs were labeled with antibodies to neurofilaments (SMI-32) and to osteopontin, which are markers of αRGCs [16,25]. Each of these markers labels only a small fraction of all RGCs, yet their overlap was extensive (S1 Fig): ~77% of the YFP-positive RGCs in KCNG4-cre;thy1-stop-YFP1 mice were SMI-positive and 92% were osteopontin-positive; 80% of the SMI-32 positive neurons and 73% of the osteopontin-positive neurons were YFP positive. Together, these features suggest that the RGCs labeled in KCNG4-cre;thy1-stop-YFP1 mice are primarily αRGCs.

**Fig 1:**
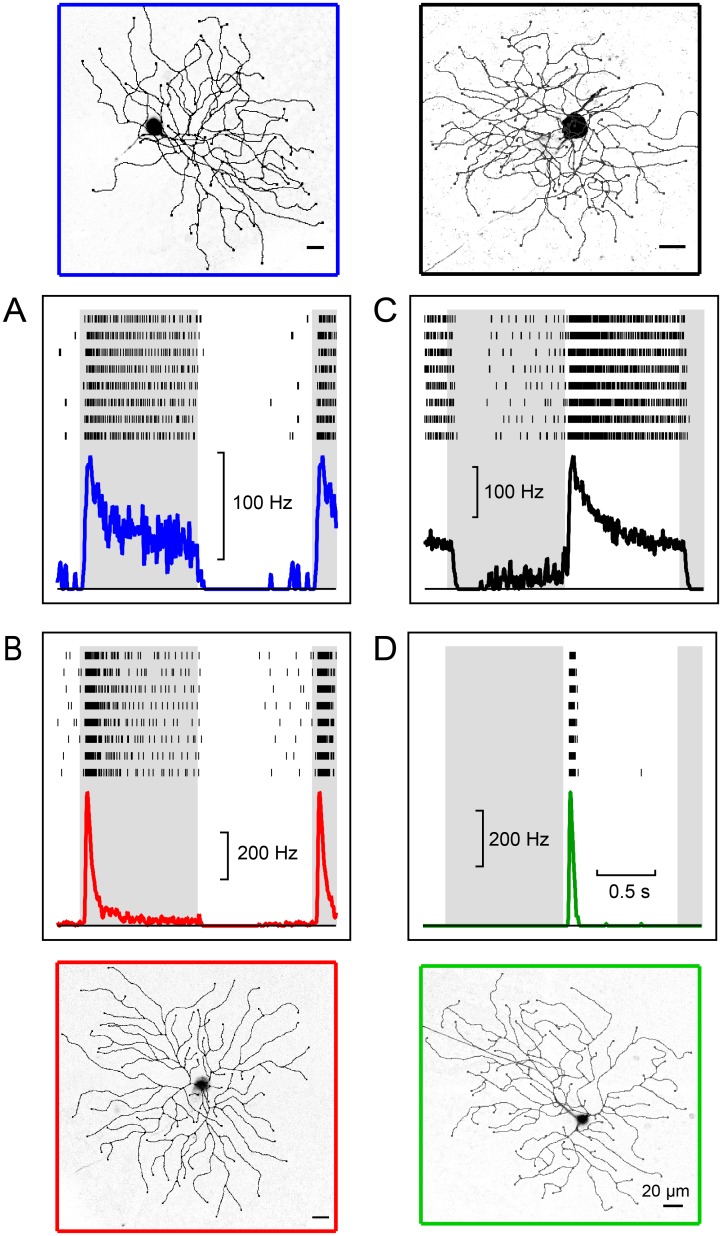
Morphology and light responses of mouse alpha retinal ganglion cells (αRGCs). A-D, Sample neurons with whole-mount views (outer images) and responses to a flashing spot (inner plots) for the Off-sustained (A), Off-transient (B), On-sustained (C), and On-transient (D) types. Raster graph illustrates action potentials on repeated trials of a spot flashing on (white background) and off (gray) every 2 s. Continuous curve is average firing rate over 10 or more trials.

To test that notion further we targeted single fluorescent ganglion cells for electrical recording and subsequently filled them with neurobiotin to inspect their structure. Results from four sample neurons are illustrated in Fig 1. Each cell was presented with the same visual stimulus: a circular disk centered on the cell body flashing black and white on a gray background. Four kinds of light response were observed: Off-sustained, with maintained firing during the dark phase and little or no firing during the bright phase (Fig 1A); Off-transient, with a burst of spikes at the start of the dark phase followed by rapid decay to little or no firing (Fig 1B); On-sustained, with maintained firing during the bright phase (Fig 1C); and On-transient, with a brief burst at the start of the bright phase (Fig 1D). The whole-mount views of these filled neurons show large cell bodies and large circular dendritic trees.

**Fig 2:**
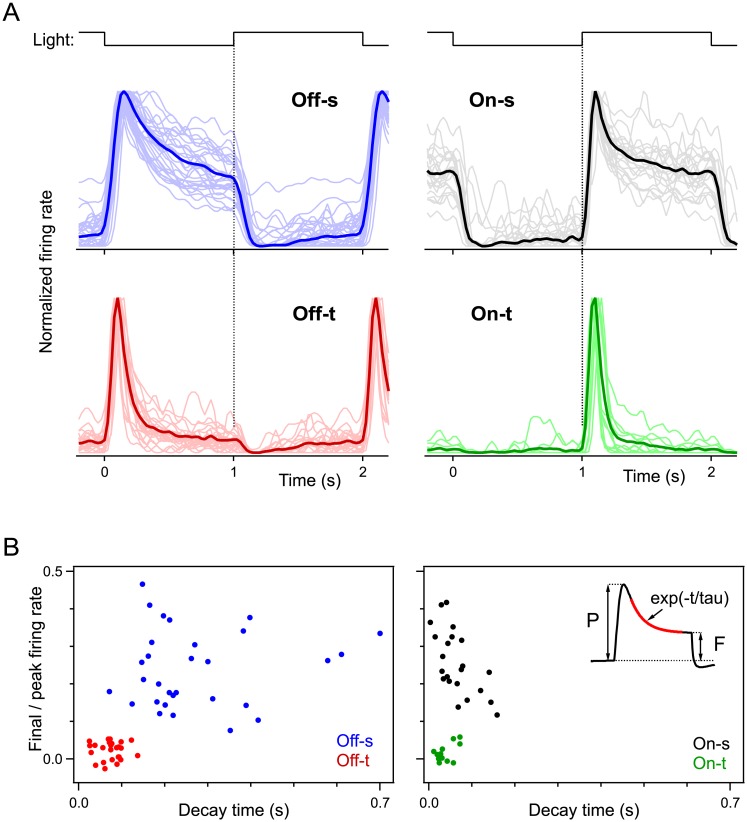
Light response kinetics define four physiological types of αRGCs. A: Time course of the firing rate during flashing spot experiments (as in Fig 1), normalized to the peak rate for each cell, and sorted by response type. Results from individual cells (faint lines) and their mean (bold). B: Scatter plot of response parameters for all αRGCs analyzed. For each cell the time course was approximated with an exponential decay (see inset). The abscissa shows the time constant of the decay, and the ordinate plots the ratio of final value to peak value of the firing rate. Among 91 alpha cells recorded by this targeting method we encountered 26% Off-t, 30% Off-s, 13% On-t, and 22% On-s.

Inspection of the entire population of recorded neurons revealed only these four functional types (Fig 2). The dynamics of light responses to the flashing spot separated clearly into On- and Off-polarity (Fig 2A). Within each polarity, one group of cells fired only briefly after the transition, whereas the other group maintained a firing rate that decayed gently from the initial peak to a steady level. We analyzed these features further by measuring for each neuron’s response the peak firing rate and the exponential decay time of the subsequent decline. Scatter plots of these response parameters showed two well-separated clusters of Off cells and another two clusters of On cells (Fig 2B). Based on these graphs we therefore identified four functional types: Off-s, Off-t, On-s, or On-t. These names will be used in the remainder of the report.

**Fig 3:**
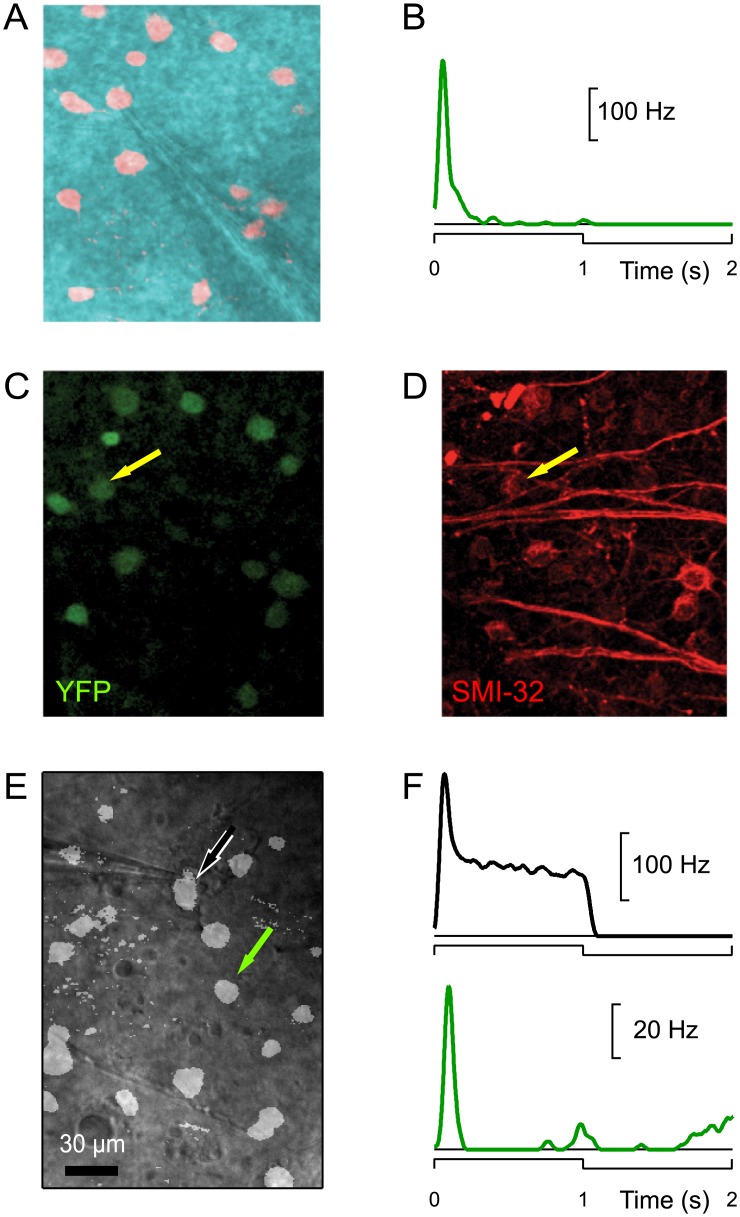
Confirmation that On-transient KCNG4-cre neurons are a separate αRGC type. A-D: On-t cells express heavy neurofilament. A loose patch recording of a fluorescent neuron (A) revealed a transient response of the firing rate to light steps (B). After fixation and antibody staining one can identify the same cell based on YFP label (C) and confirm that it is strongly labeled with the neurofilament antibody SMI-32 (D). E-F: Two fluorescent neurons in close proximity (black and green arrowheads in **E**) showed sustained (black) and transient (green) response of the firing rate to a light step (**F**).

The first three of these αRGC types have been described in prior studies of the mouse retina [13,14], but the On-transient type is new. This raised the concern whether it truly belongs to the conventionally defined alpha class, or represents some quirk of expression in the KCNG4-Cre line. To test for the classic neurofilament label, we identified On-t cells by electrical recording from fluorescent neurons, then stained the retina using SMI-32 antibody, and identified the recorded neuron in the stained tissue. Of three On-t cells tested in this way all were positive for SMI-32 (Fig 3A–3D). As elaborated below, the On-t cells share additional physiological features with the three conventional αRGC types. Thus we conclude that the On-transient neurons in the KCNG4-Cre line are αRGCs by all the criteria that define that class. Another concern was whether the difference between the On-t and On-s responses (Fig 2) might somehow arise as an artifact, owing to variations in the physiological conditions of different retina preparations. Speaking against this interpretation is the observation of both sustained and transient On-responses in the same retina and among near-neighboring αRGCs (Fig 3E and 3F).

### Structural and functional organization of the four αRGC types

**Fig 4:**
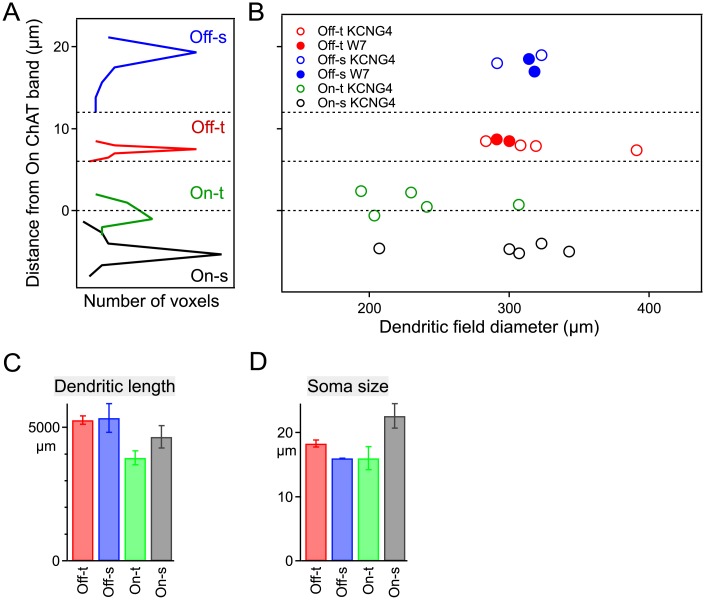
The four αRGC types stratify their dendrites in distinct layers of the IPL. A: Stratification of the 4 neurons from Fig 1. Each histogram indicates the depth distribution of fluorescent voxels in the dendrites of one cell relative to the two ChAT bands in the inner plexiform layer (at 0 and 12 μm depth). **B:** Stratification and dendritic diameter of many αRGCs, visualized either using the KCNG4-Cre line (all types) or the W7 transgenic line (Off types). For each cell, the stratification level is the mean of the histogram computed as in panel a. C-D: Total dendritic length (C) and soma diameter (D) for the four αRGC types; mean ± SEM (n = 7, 4, 6, 5 left to right in each bar graph).

We analyzed the dendritic arbors of αRGCs, both in a planar view and in depth. The inner plexiform layer of the retina is precisely organized, and the synapses of different bipolar and amacrine types are restricted to specific levels within the IPL [26–28]. Thus the stratification level of a ganglion cell’s dendrites sets constraints on what signals it can receive. All of the αRGCs we inspected were monostratified in narrow bands within the IPL (Fig 4A and 4B). As expected, the Off types stratified in the outer portion of the IPL, and the On types in the inner portion. Using the two bands of choline acetyltransferase (ChAT) expression as a reference, we confirmed the previously reported stratification levels of the Off-s, Off-t, and On-s types [14]. The new On-t type stratified in close proximity to the inner ChAT band, mirroring the Off-t type located just inside the outer ChAT band. In planar view, the four αRGC types were less distinct. They all had large dendritic fields of ~300 μm diameter (Fig 4B), with the On-t cells slightly smaller on average. All four αRGC types had a similar total length of dendrites (Fig 4C), and large soma diameters >15 μm (Fig 4D). For comparison, the dendritic field diameters of other RGC types are: W3b, 130 μm; F-midi, 175 μm; J-RGC, 240 μm; BD, 260 μm [19,29]. Thus, αRGCs are among the largest RGC types identified in mouse.

**Fig 5:**
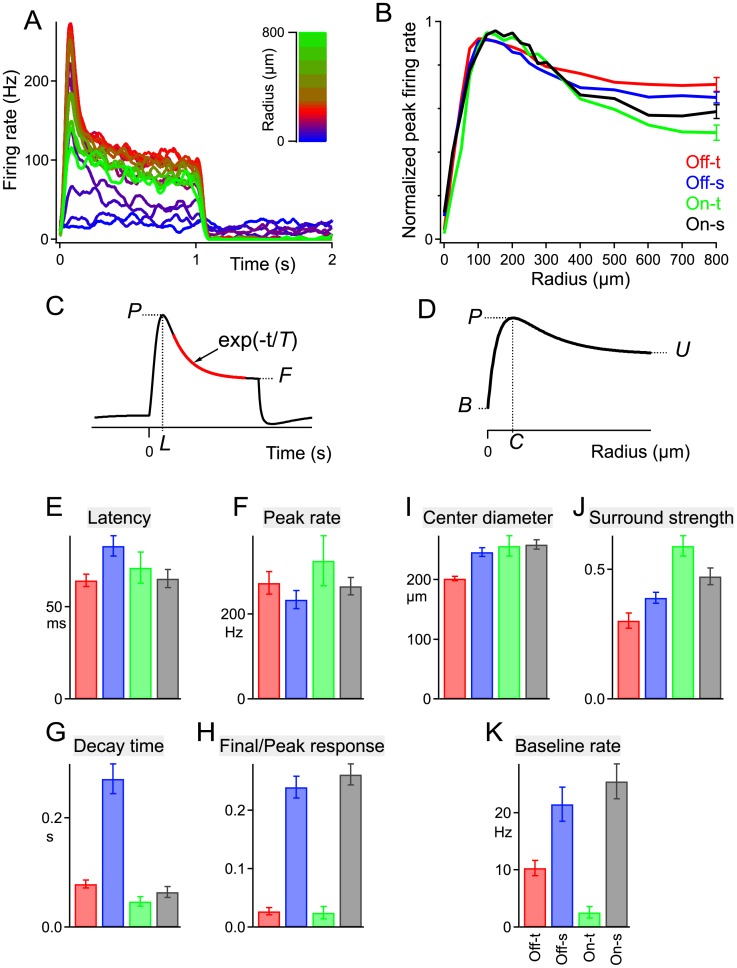
Spatio-temporal responses of the four αRGC types. A: Responses of a sample On-s cell to flashing spots of increasing radius (see inset color scale). Firing rate averaged over 6 repeats. **B:** Peak firing rate as a function of spot size from experiments such as in panel a. Curves were normalized to the maximal rate for each cell, then averaged over all cells of the same type. The error bars on the last data point are representative for SEM throughout the curve. C: From the spot stimulus giving the strongest response in panel a, one derives the latency to peak firing (*L*), the peak firing rate (*P*), the final firing rate (*F*) and the exponential decay time (*T*), as indicated in this schematic. D: From the measurements of panel B, one defines the baseline firing rate (*B*), the spot size producing the maximal rate (*C*) and the response to large uniform stimuli (*U*), as indicated in this schematic. **E-K:** Response parameters of all 4 cell types. Mean ± SEM over all cells of the same type (n = 28, 32, 8, 22 left to right in all bar graphs). Based on the measures from panels c and d: Latency = *L*; Peak rate = *P*; Center diameter = *C*; Surround strength = 1-(*U*-*B*)/(*P*-*B*); Decay time = *T*; Final/Peak response = (*F*-*B*)/(*P*-*B*); Baseline rate = *B*.

We probed the receptive fields of αRGCs using the conventional spot series method: A small circular spot centered on the ganglion cell body was flashed on and off periodically; then the spot size was gradually increased. The response was measured by the peak firing rate following the On or Off steps (Fig 5A). With increasing spot size, the response increased, reached a maximum and then declined (Fig 5B). The spot eliciting the largest response was taken as covering the receptive field center (Fig 5D). The size of this center region was similar for all four αRGC types: ~200–250 μm in diameter, slightly smaller than the dendritic fields (Fig 5I). The receptive field surround had a modest effect, producing a response suppression of ~40% from the peak value obtained with center-only stimulation (Fig 5D and 5J). Again, the four types were rather similar in this respect.

The dynamics of the light response were assessed by inspecting the time course of the firing rate under a flashing spot stimulus of optimal size (Fig 5C). The response latency, from the light step to the peak rate of firing, was very similar across the four types (Fig 5E). They also all reached the same peak firing rate of ~250 Hz (Fig 5F). The subsequent relaxation from the peak happened very quickly (~50 ms decay time) in all the αRGC types except for Off-s (~250 ms), which stood out clearly in this regard (Fig 5G).

Comparing the final firing rate long after the light step to the peak firing rate, there was a dramatic difference between sustained and transient types (Fig 5H). This is unsurprising, because that feature served to define the types in the first place (Fig 2B). However, we found a similar difference in their baseline firing rates observed under a steady gray illumination (Fig 5K), suggesting that the sustained types receive synaptic inputs that are more tonically active even under constant light.

**Fig 6:**
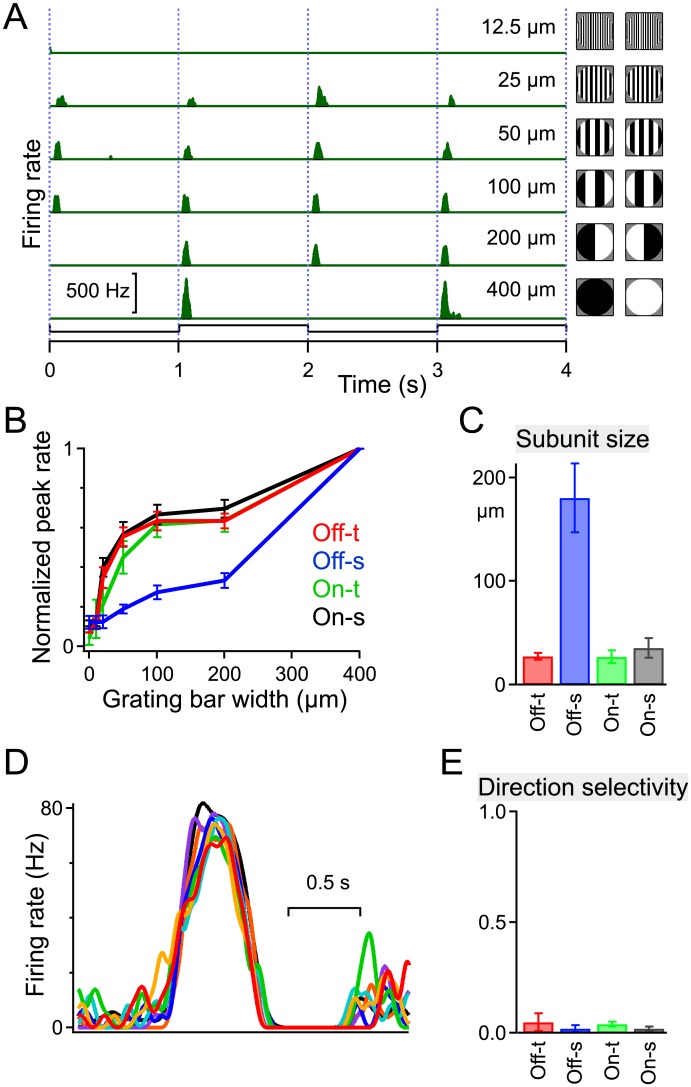
Nonlinear subunits and directional processing. A-C: Tests for nonlinear summation within the receptive field. The stimulus was a 400 μm-diameter spot centered on the receptive field, filled with a stripe grating that contrast-reversed every 1 s. For stripes of width 200 μm or less, a stripe boundary passed through the spot center. **A:** A sample RGC fires a burst of spikes on every grating transition unless the stripe width drops below a threshold, here 25 μm. **B:** Peak firing rate as a function of stripe width, normalized to the response to the uniform spot (400 μm); mean ± SEM across cells of each type. **C:** Threshold stripe width, an estimate of subunit size; mean ± SEM across cells of each type (n = 22, 23, 8, 20 left to right in panels B and C). All alpha types except Off-s show nonlinear summation over subunits ~30 μm in size. **D-E:** Tests of direction selectivity. **D:** Firing of a sample RGC in response to a 250 μm diameter spot of the preferred polarity moving through the receptive field center at 700 μm/s in 8 directions spaced at 45° (different colors). **E:** Direction selectivity index computed from such responses as
$$D=\left|\sum_{k}P_{k}e^{i\varphi_{k}}/\sum_{k}P_{k}\right|,$$
where *φ*_*k*_ is the direction of motion of the *k*-th stimulus, and *P*_*k*_ is the peak firing rate evoked by that stimulus. Mean ± SEM across cells of each type (n = 3, 3, 7, 3 left to right).

We also tested for a nonlinear subunit structure within the receptive field [9], by covering the receptive field center with a square grating that contrast-reversed periodically (Fig 6A and 6B). While making the grating progressively finer we noted the stripe width at which a response was barely detectable. For all but the Off-s type this threshold occurred at stripes of ~30 μm width (Fig 6C). This suggests that the ganglion cell receives rectified input from bipolar cells with a receptive field of ~30 μm diameter, 10 times smaller than the receptive field center of the ganglion cell. However, the Off-s type behaved very differently and revealed little nonlinear input on a scale smaller than the receptive field center. Together with the exceptionally slow decay of its step response (Fig 5G) this suggests that the Off-s ganglion cell collects input from a presynaptic circuitry different from the rest of the αRGC types.

Finally, we tested responses to moving spots traveling through the receptive field center (Fig 6D). All αRGC types responded well to such a moving stimulus, and did so equally for all directions of motion, with no hint of direction selectivity (DSI ≤ 0.05; Fig 6E) For comparison, the DSI of the BD, F-mini, and J-RGCs are 0.36, 0.33, and 0.28, respectively [19,29].

### All four αRGC types share a distinctive action potential waveform

**Fig 7:**
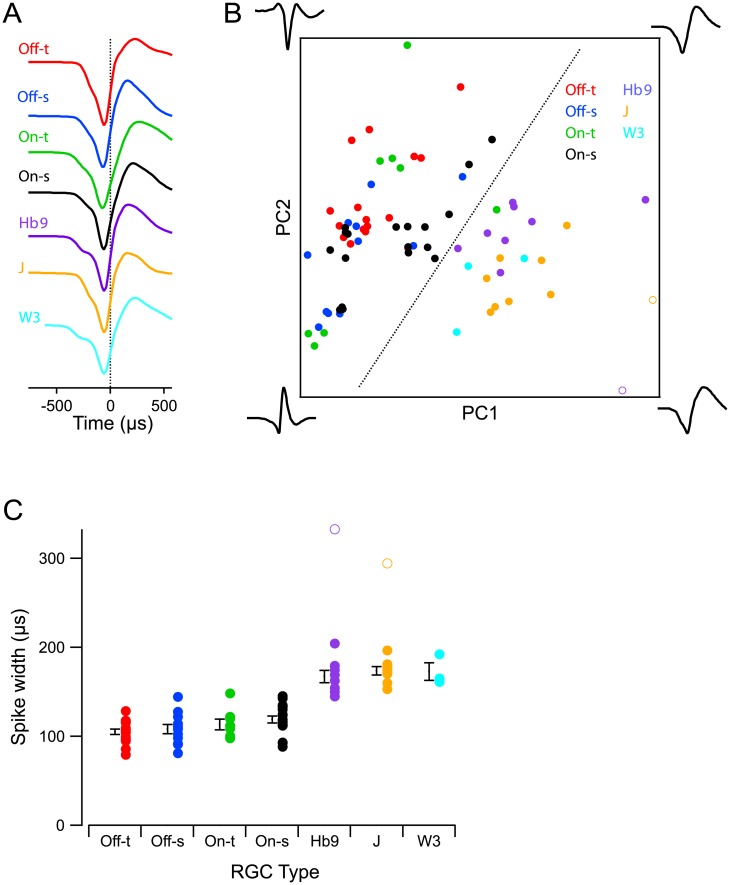
All αRGC types share a distinctive spike shape. A: The spike waveforms of seven ganglion cells, each averaged over many hundreds of spikes. These are representatives of the four αRGC types and three other identified types marked in transgenic lines: J-RGCs (J [31]), upward-coding On-Off DS cells (Hb9 [32]), and W3-RGCs (W3 [33]). All waveforms are aligned on the point with maximum time derivative. **B:** Waveform analysis of spikes from 50 RGCs of the types introduced in panel A. We computed the time derivative of each waveform, then subjected this set to a principal components analysis, and plotted the coefficients along the first two components (which accounted for 67% of the variance). Each point is one RGC’s waveform. Dotted line separates the αRGCs from all other RGCs, with just one exception. Corners of the plot are marked with the spike waveforms (width 2 ms) corresponding to those points in principal components space. **C:** Spike width—defined as the time between points of minimum and maximum slope of the action potential—for cells of the different types. Bars indicate Mean ± SEM for each cell type (n = 16, 12, 8, 18, 8, 8, 3 left to right). Two outliers (marked with open symbols in panels B and C) were excluded from this spike width analysis.

As a further signature of cell physiology we analyzed the waveform of the action potential obtained from these loose-patch recordings. In general this spike waveform reflects the combination of ionic conductances in the membrane as well as the geometry of the cell and its electrical compartments [30]. We compared spikes from the four αRGC types to those of three other genetically identified RGC types, with cell sizes ranging from small (W3 RGCs) to medium (J-RGCs) and large (On-Off DS cells). The αRGCs had spike shapes that appeared notably different from those of the other neurons (Fig 7A), as confirmed in a non-parametric shape analysis (Fig 7B). Specifically the negative phase of the action potential was considerably shorter in αRGCs (Fig 7B and 7C). Note this phase corresponds to the period of current inflow at the soma. There was no evidence for systematic differences among the αRGC types (Fig 7B and 7C). This suggests that they share a common membrane physiology that supports a fast action potential and is at least quantitatively distinct from that of other prominent RGC types.

The shared spike shape across the four αRGC types is a further indication that they belong to the same class. As a practical application, this spike shape may serve to identify αRGCs in extracellular recordings, much as “fast-spiking” and “regular-spiking” neurons are distinguished in other brain regions [30].

### The four αRGC types have distinct molecular signatures

The four αRGC types described above share three molecular features that distinguish them from most or all non-alpha cells: expression of KCNG4-cre (as judged by labeling in the KCNG4-cre line) and high levels of osteopontin and neurofilaments [16]. On the other hand, they each have distinct morphological and physiological features. This suggests that they are likely to be molecularly distinct as well. To seek such distinctions, we adopted a candidate marker approach, using osteopontin as a fiducial marker that labels αRGCs. We stained retinal sections or whole mounts with antibodies to osteopontin plus the candidate, then focused on markers present in some but not all the osteopontin-positive cells.

**Fig 8:**
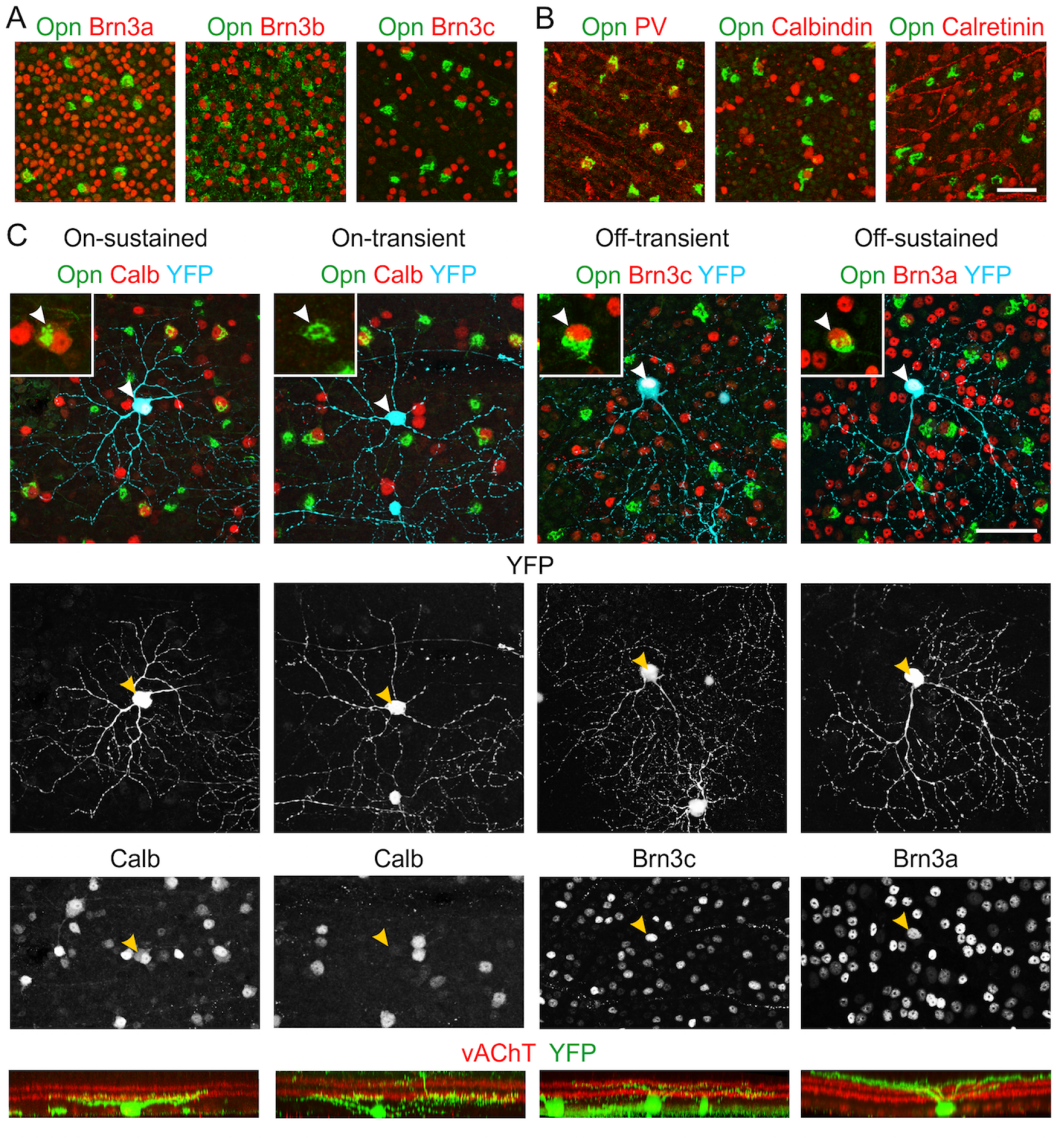
Molecular distinctions among αRGCs. **A, B**: Retinal whole mounts were stained with antibodies to Opn (green), plus antibodies to one of 3 POU-domain transcription factors (Brn3a, Brn3b or Brn3c; red in **A**) or one of 3 calcium binding proteins (parvalbumin [PV], calbindin or calretinin; red in **B**). Brn3b and PV mark most αRGCs whereas Brn3a, Brn3c and calbindin mark subsets; most αRGCs are calretinin-negative. **C:** Whole mounts of YFP-H retina were quadruply stained for YFP, Opn, vAChT and the indicated marker. Cells that were Opn, YFP, and marker triple-positive were identified (green, cyan and red, respectively in top panels) and imaged (YFP only, middle panels). Stratification of YFP-positive dendrites was then determined with reference to that of starburst amacrines (vAChT-positive, red in bottom panels). Arrows point to the same cell displayed in each panel. Results are representative of 7–10 cells per type from 5 mice. Scale bar = 50 μm.

Two groups of proteins proved to be useful in this context. The first were three related transcription factors, Brn3a, b, and c (Pou4F1-3), which are known to be differentially and combinatorially expressed by RGC subsets [34,35]. Brn3b appeared to be expressed in a majority of αRGCs (67±5% of Opn-positive cells, mean ± SEM across 3–5 fields in 2 retinas), Brn3a was expressed in about half (48±4%) and Brn3c was expressed in about one-quarter (25±3%) of αRGCs (Fig 8A). The second was a set of three calcium binding proteins that are differentially and combinatorially expressed by RGC subsets (Fig 8B): parvalbumin (PV), calbindin, and calretinin [36–38]. PV was present in most αRGCs (73±4%), consistent with previous results [36,39], calbindin was found in a minority (27±2%), and only few αRGCs were calretinin-positive (13±4%). We also found that the ON-sustained alphas stained weakly for melanopsin, as described previously [40]. In addition, we found some osteopontin-positive RGCs that stained more strongly for melanopsin, raising the possibility that some intrinsically photosensitive RGCs of the M1 or M2 class are positive for KCNG4 and osteopontin. However, we did not study these cells further.

Based on these results, we analyzed the morphology of the Brn3a-, Brn3c-, and calbindin-positive αRGCs. We used a mouse line, Thy1-YFP-H, in which YFP is expressed in a small set of RGCs [41]. Labeling is sufficiently sparse to permit clear visualization of dendritic morphology (~200 RGCs per retina [42]) and includes a wide variety of RGC types [43]. We triple-stained whole mounts with antibodies to osteopontin, GFP (which recognizes YFP) and the candidate, then imaged individual RGCs. This analysis demonstrated that among αRGCs, Brn3a is present in all Off cells but is dim or absent in On cells; Brn3c is present only in Off-t cells, and calbindin is present only in On-s cells (Fig 8C). Thus, two of the four αRGC types can be identified by a two-gene signature: Off-t αRGCs are OPN^+^Brn3c^+^, and On-s αRGCs are OPN^+^Calbindin^+^. At present three genes are required to identify Off-s and On-t αRGCs: they are OPN^+^Brn3a^+^Brn3c^-^ and OPN^+^Brn3a^-/dim^Calbindin^-^, respectively.

### Transgenic lines label subsets of αRGCs

Previous studies have reported that two transgenic mouse lines, TYW7 [19] and CB2-GFP [21], label subsets of αRGCs. These two lines express YFP (TYW7) or GFP (CB2) under control of Thy1 and calbindin 2 (calretinin) regulatory elements, respectively, with patterns of expression that differ from those of the endogenous gene, presumably due to effects of genomic elements near the site of transgene integration [41]. We asked whether these αRGCs were included within the types labeled by KCNG4, or whether they represented additional populations.

**Fig 9:**
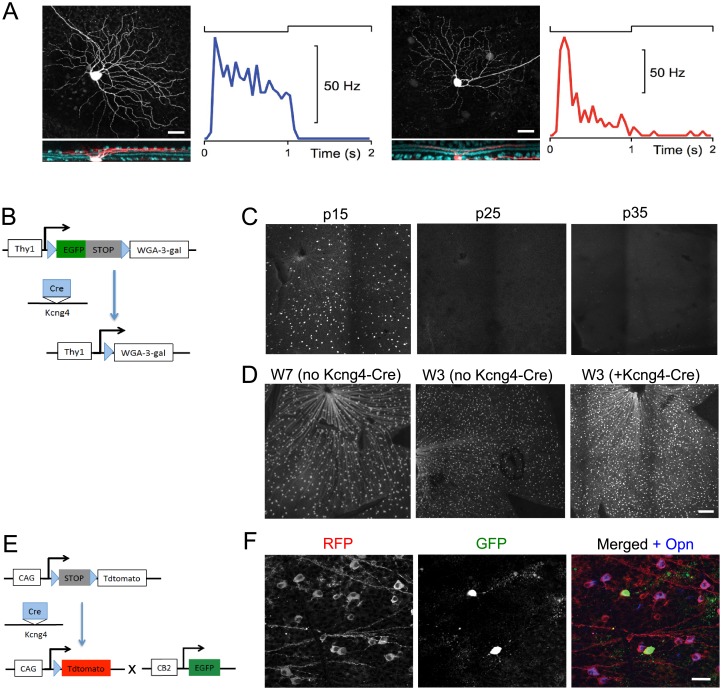
Existing transgenic lines label subsets of αRGCs. **A:** Morphology and physiology of YFP-positive cells in the TYW7 line. Structure and function were assessed as in Fig 1. The cell shapes (top: whole mount view, bottom: vertical projection including ChAT label) and the light responses (firing rate under periodically flashing spot) identify these as Off-s (left) and Off-t (right) αRGCs. Of n = 10 cells recorded in this line, 5 were Off-s and 5 were Off-t. **B:** Use of the cre-off feature of the TYW3 and TYW7 lines: YFP is flanked by lox sites in these lines, so it is excised in cells that also express cre. **C:** YFP disappears with age from retinas in the TYW7; Kcng4-cre double transgenics at the indicated postnatal ages. Therefore the TYW7 line labels a subset of αRGCs. **D:** YFP persists in TYW3; Kcng4-cre double transgenics. Therefore the TYW3 line labels a set of non-αRGCs. **E:** Triple transgenic strategy to label both Kcng4-cre neurons (RFP) and CB2-GFP neurons (GFP). **F:** In these triple transgenics, the CB2-GFP-positive RGCs also express Kcng4-cre and osteopontin (Opn). Therefore the CB2 line labels a subset of αRGCs. Scale bars: (A) 20 μm, (C and D) 200 μm, (F) 20 μm.

Morphological and physiological analyses revealed that YFP-positive cells in the TYW7 line were of two types, corresponding to Off-t and Off-s αRGC (Fig 9A). To determine whether these are the same types labeled in the KCNG4-cre line, we made use of the fact that TYW7 is a “cre-off” line in which expression of cre leads to loss of YFP (Fig 9B [19]). We crossed TYW7 mice to KCNG4-Cre and asked whether expression of YFP was extinguished. YFP expression was attenuated in KCNG4-Cre;TYW7 retinas by postnatal day 15 (P15), shortly after cre is activated in KCNG4-cre mice (~P12); the loss was dramatic by P25 and complete by P35 (Fig 9C). No loss of YFP was seen when KCNG4-Cre was crossed to a similarly constructed line, TYW3, which labels a distinct subset of RGCs [19,33,44], indicating the specificity of the effect (Fig 9D).

Huberman et al. (2008) demonstrated that the CB2-GFP line labels Off-t αRGCs. To ask whether these cells were included among KCNG4-Cre αRGCs, we analyzed triple transgenic mice in which KCNG4-cre-expressing cells were labeled with a red fluorescent proteins (CB2-GFP; KCNG4-Cre; Rosa-CAG-STOP-RFP; Fig 9E). All GFP-positive neurons were also RFP positive, demonstrating that CB2-GFP-labeled cells were also KCNG4-cre-positive (Fig 9F).

Together these results indicate that the αRGCs labeled in KCNG4-cre mice include those labeled in TYW7 and CB2-GFP mice, consistent with our hypothesis that the KCNG4-cre line labels all αRGCs, and that all αRGCs are osteopontin-positive.

### Mosaic analysis of αRGC types

**Fig 10:**
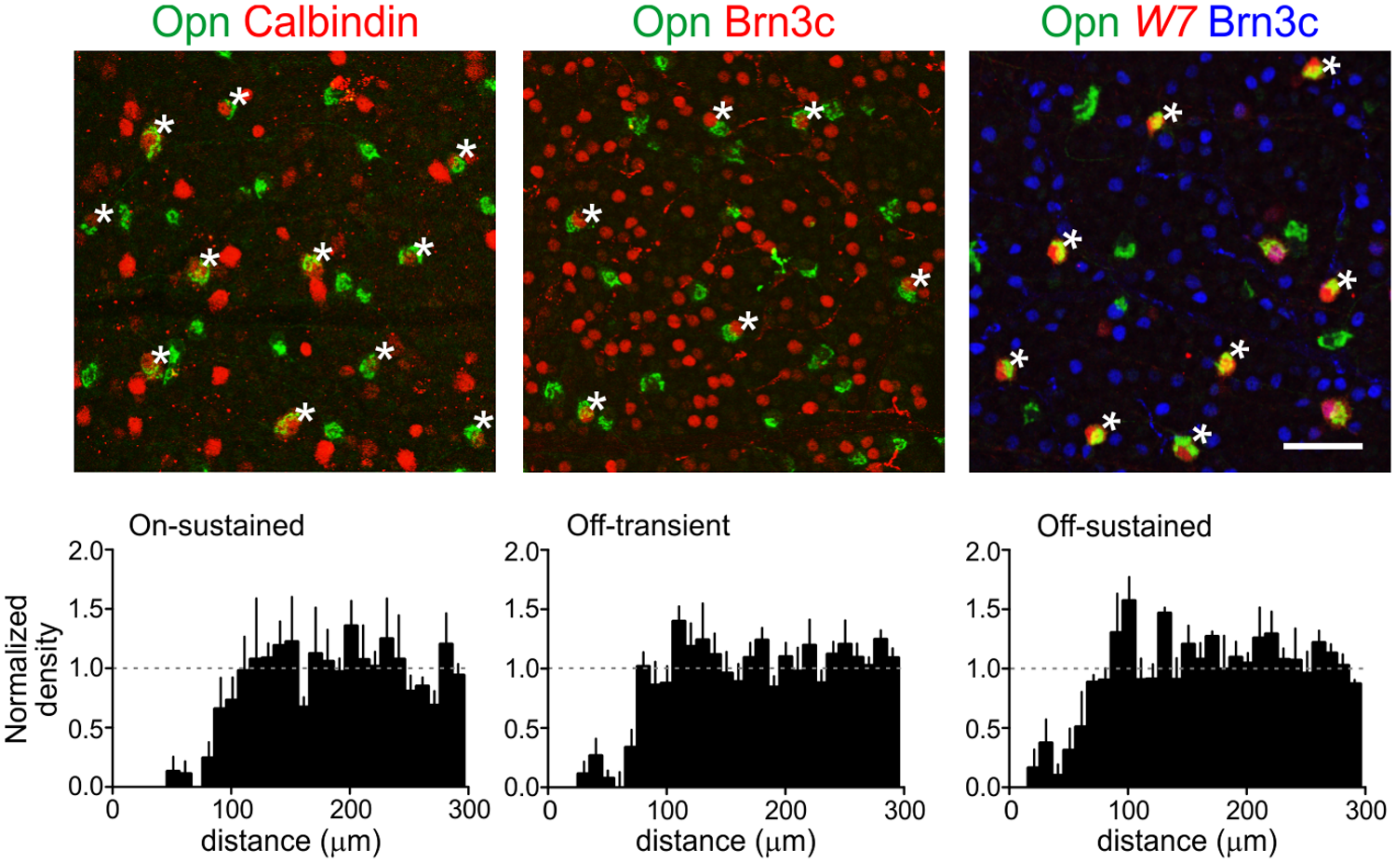
Mosaic organization of αRGC types. Three types of αRGCs were distinguished in retinal whole mounts by the molecular markers indicated above the top panels (see text and Fig 8 for details; asterisks show cells of the indicated type). The TYW7 line was used instead of Brn3a to label all Off αRGCs, because of species incompatibilities of antibodies; the Off-s αRGCs are W7 positive but Brn3c negative. For the On-t cells, we lacked a combination of markers to label them with sufficient reliability. A density recovery profile (DRP) analysis was then performed on each αRGC type (bottom panels; n = 3–5 retinas per type from 3 mice). The prominent dip in density at short distances is characteristic of “repulsion” between cell bodies of the same type. Dashed line, normalized average density. Mean ± SEM across cells of each type. Scale bar = 50 μm.

Can the groups of αRGCs we have described be subdivided further or are they natural cell types? In the retina, neurons of a specific type are arranged in a pattern called a mosaic, with two cells of a single type less likely to be near neighbors than they would be expected by chance [45,46]. In contrast, cells of different types are arranged randomly with respect to each other. Thus, mosaic spacing of the cell bodies, as assessed by density recovery profile (DRP) analysis [33,47], provides a means of testing whether a population comprises a single type, multiple types, or only part of a type. We therefore performed DRP analysis on the three αRGC types for which we had unique molecular signatures, using the markers described above and the TYW7 line. In each case, we found clear “repulsion” among the neurons with the same molecular markers, indicating that they each form a unique mosaic (Fig 10). At present the On-transient type can only be identified with three coincident markers, and the resulting experimental variation led to excessive noise in the DRP analysis.

## Discussion

**Fig 11:**
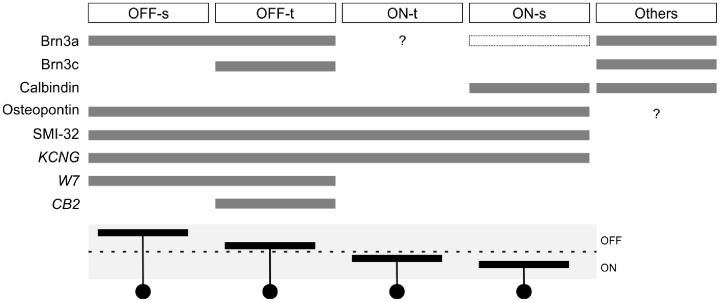
Morphological, physiological and molecular properties distinguish four αRGC types. A summary of morphological, physiological and molecular features for the four types of αRGCs, as reported in Figs 2, 4 and 8–10. Dotted box indicates dim labeling. See Discussion.

This study extends our understanding of αRGCs, which form perhaps the fastest pathway for visual information to reach the brain. We showed here that αRGCs in the mouse express a shared set of molecular markers that facilitate their targeted study (Fig 3 and [16]) and a shared physiological feature, the shape of the action potential, that distinguishes them from other types of ganglion cells (Fig 7). Most importantly, we show that there exist not three but four types of αRGCs and that they cover the space of both function and morphology in a beautifully symmetric arrangement (Figs 1, 2, 4, 5 and 6). Finally we identify specific genetic markers that allow for the identification of three out of four of these types (Figs 8–11).

### Four αRGC types in the mouse retina

By recording from αRGCs in the KCNG4-Cre transgenic mouse line, we found that they fall into four functional and morphological types: two On types and two Off types, with one member of each pair producing transient and the other sustained responses (Figs 2–4). All four types have narrowly stratified dendritic trees. As expected, the On types ramify in the inner lamina of the IPL and the Off types in the outer lamina. The transient types ramify inside the two ChAT bands, and the sustained types just outside the ChAT bands (Fig 4), reinforcing prior observations on transient and sustained zones of the IPL [26,48]. Altogether, these four cell types exhibit a remarkable symmetry with respect to polarity of the light responses, their kinetics, and their morphological arrangement.

Three of these αRGC types had been observed before [13,14], but the On-transient type is new, at least in its recognition as an αRGC. This type may have been missed because it is somewhat less numerous and has a slightly smaller dendritic field than the On-sustained type (Figs 2, 4B and 4C). However, broad unbiased surveys of the RGC population include cell types that likely correspond to the On-transient αRGC. In the survey of dendritic morphology by Sümbül et al [28] one finds a cluster “X” with monostratified arbors just distal to the On-ChAT band, measuring 200–250 μm in diameter. The electron-microscopic reconstructions of Helmstaedter et al [49] also revealed a cluster “gc47-57” that ramifies at this location. These RGCs receive direct bipolar contacts mostly from type 5 cone bipolars, known to have very transient light responses [26], which accords well with the function of On-t cells reported here. Another possible match, in terms of both response kinetics and stratification, is the “PV2” cell reported by Farrow et al [36]. Finally the recent calcium imaging survey of Baden et al [50] reports a type “G19”, described as “On transient large”, which also stains with the neurofilament antibody SMI-32. We suggest that all these “orphan” RGC types correspond to the On-transient alpha cell studied in the present report.

Aside from the obvious differences in response kinetics, the four alpha types have very similar structure and function (Figs 4–7). Only the Off-sustained cell deviates in two ways: It shows weak non-linear summation (Fig 6C), and its firing rate decays very slowly from the peak (Fig 5G). It has been suggested that Off-sustained αRGCs are modulated not by changes in excitation, but primarily via glycinergic inhibition from the On channel [14,51]. Furthermore they receive synaptic input from an unusual monopolar interneuron [52]. These differences in presynaptic circuitry may explain why the Off-sustained cell departs from other αRGC types in kinetics and nonlinearity.

How do these new findings in the mouse relate to αRGCs in other species? The founding reports on cat retina identified two αRGC types that carry transient signals and ramify in the central IPL [4,8,53], much like the On-transient and Off- transient cells of the mouse. Two additional types with large dendritic fields, called delta and epsilon cells in the cat, ramify close to the margins of the IPL [54]. These might be candidates for correspondence with the On-sustained and Off-sustained cells in the mouse. In the guinea pig, four RGC types with large cell bodies have been reported [51], whose dendritic stratification matches exactly that described here (compare Fig 4B here to Fig 2D of [51]). As in the mouse, they produce (from outer to inner IPL) Off-sustained, Off-transient, On-transient, and On-sustained responses. In the rat and the rabbit, the RGCs with large cell bodies and dendritic fields (“Class 1”) again divide into four morphological types, that stratify at the same positions as described here for the mouse [12,55]. Thus one is led to see a common arrangement across species, in which the large-bodied RGCs form four types that split the visual signal into channels conveying On vs Off and sustained vs transient signals. As we showed here, these four types belong together also by other criteria, including molecular markers and a distinct physiological spiking signature. It will be interesting to test this extended correspondence in other mammalian species.

### Molecular distinctions

Molecular analysis of αRGCs has revealed shared features that distinguish them from other RGCs and unique features that distinguish the four αRGC types from each other. In the first category are (a) expression of Kcng4, as inferred from activity of Cre recombinase within the endogenous *Kcng4* locus; (b) high levels of osteopontin; and (c) high levels of neurofilament proteins, best revealed by staining with the monoclonal antibody, SMI-32. Although none of these markers is entirely specific for αRGCs, all three are selective to varying degrees. In particular, we found that the SMI-32 antibody labeled αRGCs of all types, whereas a previous study used it to single out On-s and Off-t cells [56]. However, a direct comparison of the underlying micrographs (e.g. S1 Fig here and Fig 2B of [56]) shows general agreement, in that SMI-32 labels cell bodies with a graded range of intensities, and the brightest ones may well correspond to the On-s subset of αRGCs.

Might these shared molecular markers provide insight into the form or function of αRGCs? Neurofilaments are cytoskeletal elements and their expression level is an essential determinant of axonal caliber, which in turn influences conduction velocity [57,58]. Thus, the high neurofilament content of αRGCs is likely causally related to their having the largest caliber axons of all RGCs. Osteopontin is a phosphoprotein implicated in regulating a variety of cellular processes, including growth and proliferation [59]. Ectopic expression of osteopontin in non-alpha RGCs leads to an increase in their size, suggesting that osteopontin contributes to the large size of αRGCs, although genetic studies indicate that it is not the sole determinant [16]. Roles of Kcng4 are more elusive, but Müller et al. [60] recently demonstrated that Kcng4 is selectively expressed by fast motoneurons (which have larger caliber axons than slow motoneurons) and, when over-expressed, modulates action potential properties, raising the possibility that selective Kcng4 expression mediates some of the unusual firing properties of αRGCs.

Combinatorial expression of several other genes provides a way to distinguish αRGC types from each other. Three types have distinct expression patterns of the *Pou4f* (*Brn3*) genes: On-s are Brn3a^-^Brn3b^+^Brn3c^-^, Off-s are Brn3a^+^Brn3b^+^Brn3c^-^, and Off-t are Brn3a^+^Brn3b^+^Brn3c^+^. On-s and On-t αRGCs are distinguished by expression of calbindin, positive and negative, respectively. Thus, combined with an alpha “family” identifier, these genes offer a relatively simple signature that discriminates among alpha types. These markers may help in further global analysis of these ganglion cell types, for example to examine their distribution across the retina [29,33,56] or the arrangement of their terminals in retinorecipient structures.

## Supporting information

S1 Fig Three markers expressed in alpha ganglion cells. Retina of a KCNG4-cre;thy1-stop-YFP1 mouse stained with antibodies for GFP (KCNG), osteopontin (Opn), and neurofilament (SMI-32), with overlap shown in false color (KCNG Opn SMI-32). Note the strong correspondence among the 3 labels. However, because each of the markers varies somewhat in strength from cell to cell, a binary assignment to “positive” and “negative” necessarily leads to overlap numbers less than 100%, as quoted in the text.
(TIFF)

## References

[1] Roska M, Meister M (2014) The Retina Dissects the Visual Scene into Distinct Features In: Werner JS, Chalupa LM, editors. The New Visual Neurosciences. Cambridge, MA: MIT Press pp. 163–182.

[2] Sanes JR, Masland RH (2015) The types of retinal ganglion cells: current status and implications for neuronal classification. Annu Rev Neurosci 38: 221–246. doi: 10.1146/annurev-neuro-071714-034120 25897874 10.1146/annurev-neuro-071714-034120

[3] Berson DM (2008) Retinal ganglion cell types and their central projections In: Basbaum AI, Kaneko A, Shepherd GM, Westheimer G, editors. The senses: a comprehensive reference, Vol 1 San Diego: Academic Press pp. 491–520.

[4] Boycott BB, Wässle H (1974) The morphological types of ganglion cells of the domestic cat’s retina. J Physiol 240: 397–419. 4422168 10.1113/jphysiol.1974.sp010616 PMC1331022

[5] Peichl L, Ott H, Boycott BB (1987) Alpha ganglion cells in mammalian retinae. Proc R Soc Lond B Biol Sci 231: 169–197. 2889210 10.1098/rspb.1987.0040

[6] Cleland BG, Levick WR (1974) Brisk and sluggish concentrically organized ganglion cells in the cat’s retina. J Physiol 240: 421–456. 4421622 10.1113/jphysiol.1974.sp010617 PMC1331023

[7] Cleland BG, Levick WR, Wässle H (1975) Physiological identification of a morphological class of cat retinal ganglion cells. J Physiol 248: 151–171. 1151804 10.1113/jphysiol.1975.sp010967 PMC1309512

[8] Peichl L, Wässle H (1981) Morphological identification of on- and off-centre brisk transient (Y) cells in the cat retina. Proc R Soc Lond B Biol Sci 212: 139–153. 6166011 10.1098/rspb.1981.0030

[9] Enroth-Cugell C, Robson JG (1966) The contrast sensitivity of retinal ganglion cells of the cat. J Physiol 187: 517–552. 16783910 10.1113/jphysiol.1966.sp008107 PMC1395960

[10] Schwartz GW, Okawa H, Dunn FA, Morgan JL, Kerschensteiner D, Wong RO et al (2012) The spatial structure of a nonlinear receptive field. Nat Neurosci 15: 1572–1580. doi: 10.1038/nn.3225 23001060 10.1038/nn.3225 PMC3517818

[11] Demb JB, Zaghloul K, Haarsma L, Sterling P (2001) Bipolar cells contribute to nonlinear spatial summation in the brisk-transient (Y) ganglion cell in mammalian retina. J Neurosci 21: 7447–7454. 11567034 10.1523/JNEUROSCI.21-19-07447.2001 PMC6762908

[12] Famiglietti EV (2004) Class I and class II ganglion cells of rabbit retina: a structural basis for X and Y (brisk) cells. J Comp Neurol 478: 323–346. doi: 10.1002/cne.20268 15384072 10.1002/cne.20268

[13] Pang JJ, Gao F, Wu SM (2003) Light-evoked excitatory and inhibitory synaptic inputs to ON and OFF alpha ganglion cells in the mouse retina. J Neurosci 23: 6063–6073. 12853425 10.1523/JNEUROSCI.23-14-06063.2003 PMC6740343

[14] van Wyk M, Wässle H, Taylor WR (2009) Receptive field properties of ON- and OFF-ganglion cells in the mouse retina. Vis Neurosci 26: 297–308. doi: 10.1017/S0952523809990137 19602302 10.1017/S0952523809990137 PMC2874828

[15] Dhande OS, Huberman AD (2014) Retinal ganglion cell maps in the brain: implications for visual processing. Curr Opin Neurobiol 24: 133–142. doi: 10.1016/j.conb.2013.08.006 24492089 10.1016/j.conb.2013.08.006 PMC4086677

[16] Duan X, Qiao M, Bei F, Kim IJ, He Z, Sanes JR (2015) Subtype-specific regeneration of retinal ganglion cells following axotomy: Effects of osteopontin and mTOR signaling. Neuron 85: 1244–1256. doi: 10.1016/j.neuron.2015.02.017 25754821 10.1016/j.neuron.2015.02.017 PMC4391013

[17] Duan X, Krishnaswamy A, De la Huerta I, Sanes JR (2014) Type II cadherins guide assembly of a direction-selective retinal circuit. Cell 158: 793–807. doi: 10.1016/j.cell.2014.06.047 25126785 10.1016/j.cell.2014.06.047

[18] Buffelli M, Burgess RW, Feng G, Lobe CG, Lichtman JW, Sanes JR (2003) Genetic evidence that relative synaptic efficacy biases the outcome of synaptic competition. Nature 424: 430–434. doi: 10.1038/nature01844 12879071 10.1038/nature01844

[19] Kim IJ, Zhang Y, Meister M, Sanes JR (2010) Laminar restriction of retinal ganglion cell dendrites and axons: subtype-specific developmental patterns revealed with transgenic markers. J Neurosci 30: 1452–1462. doi: 10.1523/JNEUROSCI.4779-09.2010 20107072 10.1523/JNEUROSCI.4779-09.2010 PMC2822471

[20] Gong S, Zheng C, Doughty ML, Losos K, Didkovsky N, Schambra UB et al (2003) A gene expression atlas of the central nervous system based on bacterial artificial chromosomes. Nature 425: 917–925. doi: 10.1038/nature02033 14586460 10.1038/nature02033

[21] Huberman AD, Manu M, Koch SM, Susman MW, Lutz AB, Ullian EM et al (2008) Architecture and activity-mediated refinement of axonal projections from a mosaic of genetically identified retinal ganglion cells. Neuron 59: 425–438. doi: 10.1016/j.neuron.2008.07.018 18701068 10.1016/j.neuron.2008.07.018 PMC8532044

[22] Madisen L, Zwingman TA, Sunkin SM, Oh SW, Zariwala HA, Gu H et al (2010) A robust and high-throughput Cre reporting and characterization system for the whole mouse brain. Nat Neurosci 13: 133–140. doi: 10.1038/nn.2467 20023653 10.1038/nn.2467 PMC2840225

[23] Madisen L, Mao T, Koch H, Zhuo JM, Berenyi A, Fujisawa S et al (2012) A toolbox of Cre-dependent optogenetic transgenic mice for light-induced activation and silencing. Nat Neurosci 15: 793–802. doi: 10.1038/nn.3078 22446880 10.1038/nn.3078 PMC3337962

[24] Peichl L (1991) Alpha ganglion cells in mammalian retinae: common properties, species differences, and some comments on other ganglion cells. Vis Neurosci 7: 155–169. 1931799 10.1017/s0952523800011020

[25] Lin B, Wang SW, Masland RH (2004) Retinal ganglion cell type, size, and spacing can be specified independent of homotypic dendritic contacts. Neuron 43: 475–485. doi: 10.1016/j.neuron.2004.08.002 15312647 10.1016/j.neuron.2004.08.002

[26] Euler T, Haverkamp S, Schubert T, Baden T (2014) Retinal bipolar cells: elementary building blocks of vision. Nat Rev Neurosci 15: 507–519. 25158357 10.1038/nrn3783

[27] Siegert S, Scherf BG, Del Punta K, Didkovsky N, Heintz N, Roska B (2009) Genetic address book for retinal cell types. Nat Neurosci 12: 1197–1204. doi: 10.1038/nn.2370 19648912 10.1038/nn.2370

[28] Sümbül U, Song S, McCulloch K, Becker M, Lin B, Sanes JR et al (2014) A genetic and computational approach to structurally classify neuronal types. Nat Commun 5: 3512 doi: 10.1038/ncomms4512 24662602 10.1038/ncomms4512 PMC4164236

[29] Rousso DL, Qiao M, Kagan RD, Yamagata M, Palmiter RD, Sanes JR (2016) Two pairs of ON and OFF retinal ganglion cells are defined by intersectional patterns of transcription factor expression. Cell Rep 15: 1930–1944. doi: 10.1016/j.celrep.2016.04.069 27210758 10.1016/j.celrep.2016.04.069 PMC4889540

[30] Bean BP (2007) The action potential in mammalian central neurons. Nat Rev Neurosci 8: 451–465. doi: 10.1038/nrn2148 17514198 10.1038/nrn2148

[31] Kim IJ, Zhang Y, Yamagata M, Meister M, Sanes JR (2008) Molecular identification of a retinal cell type that responds to upward motion. Nature 452: 478–482. doi: 10.1038/nature06739 18368118 10.1038/nature06739

[32] Trenholm S, McLaughlin AJ, Schwab DJ, Awatramani GB (2013) Dynamic tuning of electrical and chemical synaptic transmission in a network of motion coding retinal neurons. J Neurosci 33: 14927–14938. doi: 10.1523/JNEUROSCI.0808-13.2013 24027292 10.1523/JNEUROSCI.0808-13.2013 PMC6705162

[33] Zhang Y, Kim IJ, Sanes JR, Meister M (2012) The most numerous ganglion cell type of the mouse retina is a selective feature detector. Proc Natl Acad Sci U S A 109: E2391–8. doi: 10.1073/pnas.1211547109 22891316 10.1073/pnas.1211547109 PMC3437843

[34] Badea TC, Cahill H, Ecker J, Hattar S, Nathans J (2009) Distinct roles of transcription factors brn3a and brn3b in controlling the development, morphology, and function of retinal ganglion cells. Neuron 61: 852–864. doi: 10.1016/j.neuron.2009.01.020 19323995 10.1016/j.neuron.2009.01.020 PMC2679215

[35] Badea TC, Nathans J (2011) Morphologies of mouse retinal ganglion cells expressing transcription factors Brn3a, Brn3b, and Brn3c: analysis of wild type and mutant cells using genetically-directed sparse labeling. Vision Res 51: 269–279. doi: 10.1016/j.visres.2010.08.039 20826176 10.1016/j.visres.2010.08.039 PMC3038626

[36] Farrow K, Teixeira M, Szikra T, Viney TJ, Balint K, Yonehara K et al (2013) Ambient illumination toggles a neuronal circuit switch in the retina and visual perception at cone threshold. Neuron 78: 325–338. doi: 10.1016/j.neuron.2013.02.014 23541902 10.1016/j.neuron.2013.02.014

[37] Pasteels B, Rogers J, Blachier F, Pochet R (1990) Calbindin and calretinin localization in retina from different species. Vis Neurosci 5: 1–16. 2125465 10.1017/s0952523800000031

[38] Yi CW, Yu SH, Lee ES, Lee JG, Jeon CJ (2012) Types of parvalbumin-containing retinotectal ganglion cells in mouse. Acta Histochem Cytochem 45: 201–210. doi: 10.1267/ahc.11061 22829714 10.1267/ahc.11061 PMC3394870

[39] Münch TA, da Silveira RA, Siegert S, Viney TJ, Awatramani GB, Roska B (2009) Approach sensitivity in the retina processed by a multifunctional neural circuit. Nat Neurosci 12: 1308–1316. doi: 10.1038/nn.2389 19734895 10.1038/nn.2389

[40] Estevez ME, Fogerson PM, Ilardi MC, Borghuis BG, Chan E, Weng S et al (2012) Form and function of the M4 cell, an intrinsically photosensitive retinal ganglion cell type contributing to geniculocortical vision. J Neurosci 32: 13608–13620. doi: 10.1523/JNEUROSCI.1422-12.2012 23015450 10.1523/JNEUROSCI.1422-12.2012 PMC3474539

[41] Feng G, Mellor RH, Bernstein M, Keller-Peck C, Nguyen QT, Wallace M et al (2000) Imaging neuronal subsets in transgenic mice expressing multiple spectral variants of GFP. Neuron 28: 41–51. 11086982 10.1016/s0896-6273(00)00084-2

[42] Samuel MA, Zhang Y, Meister M, Sanes JR (2011) Age-related alterations in neurons of the mouse retina. J Neurosci 31: 16033–16044. doi: 10.1523/JNEUROSCI.3580-11.2011 22049445 10.1523/JNEUROSCI.3580-11.2011 PMC3238393

[43] Coombs J, van der List D, Wang GY, Chalupa LM (2006) Morphological properties of mouse retinal ganglion cells. Neuroscience 140: 123–136. doi: 10.1016/j.neuroscience.2006.02.079 16626866 10.1016/j.neuroscience.2006.02.079

[44] Krishnaswamy A, Yamagata M, Duan X, Hong YK, Sanes JR (2015) Sidekick 2 directs formation of a retinal circuit that detects differential motion. Nature 524: 466–470. doi: 10.1038/nature14682 26287463 10.1038/nature14682 PMC4552609

[45] Reese BE, Keeley PW (2015) Design principles and developmental mechanisms underlying retinal mosaics. Biol Rev Camb Philos Soc 90: 854–876. doi: 10.1111/brv.12139 25109780 10.1111/brv.12139 PMC4320990

[46] Sanes JR, Zipursky SL (2010) Design principles of insect and vertebrate visual systems. Neuron 66: 15–36. doi: 10.1016/j.neuron.2010.01.018 20399726 10.1016/j.neuron.2010.01.018 PMC2871012

[47] Rodieck RW (1991) The density recovery profile: a method for the analysis of points in the plane applicable to retinal studies. Vis Neurosci 6: 95–111. 2049333 10.1017/s095252380001049x

[48] Roska B, Molnar A, Werblin FS (2006) Parallel processing in retinal ganglion cells: how integration of space-time patterns of excitation and inhibition form the spiking output. J Neurophysiol 95: 3810–3822. doi: 10.1152/jn.00113.2006 16510780 10.1152/jn.00113.2006

[49] Helmstaedter M, Briggman KL, Turaga SC, Jain V, Seung HS, Denk W (2013) Connectomic reconstruction of the inner plexiform layer in the mouse retina. Nature 500: 168–174. doi: 10.1038/nature12346 23925239 10.1038/nature12346

[50] Baden T, Berens P, Franke K, Roman Roson M, Bethge M, Euler T (2016) The functional diversity of retinal ganglion cells in the mouse. Nature 529: 345–350. doi: 10.1038/nature16468 26735013 10.1038/nature16468 PMC4724341

[51] Manookin MB, Beaudoin DL, Ernst ZR, Flagel LJ, Demb JB (2008) Disinhibition combines with excitation to extend the operating range of the OFF visual pathway in daylight. J Neurosci 28: 4136–4150. doi: 10.1523/JNEUROSCI.4274-07.2008 18417693 10.1523/JNEUROSCI.4274-07.2008 PMC2557439

[52] Della Santina L, Kuo SP, Yoshimatsu T, Okawa H, Suzuki SC, Hoon M et al (2016) Glutamatergic Monopolar Interneurons Provide a Novel Pathway of Excitation in the Mouse Retina. Curr Biol 26: 2070–2077. doi: 10.1016/j.cub.2016.06.016 27426514 10.1016/j.cub.2016.06.016 PMC4980212

[53] Wässle H, Boycott BB, Illing RB (1981) Morphology and mosaic of on- and off-beta cells in the cat retina and some functional considerations. Proc R Soc Lond B Biol Sci 212: 177–195. 6166013 10.1098/rspb.1981.0033

[54] Berson DM, Pu M, Famiglietti EV (1998) The zeta cell: a new ganglion cell type in cat retina. J Comp Neurol 399: 269–288. 9721908 10.1002/(sici)1096-9861(19980921)399:2<269::aid-cne9>3.0.co;2-z

[55] Peichl L (1989) Alpha and delta ganglion cells in the rat retina. J Comp Neurol 286: 120–139. doi: 10.1002/cne.902860108 2768556 10.1002/cne.902860108

[56] Bleckert A, Schwartz GW, Turner MH, Rieke F, Wong RO (2014) Visual space is represented by nonmatching topographies of distinct mouse retinal ganglion cell types. Curr Biol 24: 310–315. doi: 10.1016/j.cub.2013.12.020 24440397 10.1016/j.cub.2013.12.020 PMC3990865

[57] Lee MK, Cleveland DW (1996) Neuronal intermediate filaments. Annu Rev Neurosci 19: 187–217. doi: 10.1146/annurev.ne.19.030196.001155 8833441 10.1146/annurev.ne.19.030196.001155

[58] Muma NA, Slunt HH, Hoffman PN (1991) Postnatal increases in neurofilament gene expression correlate with the radial growth of axons. J Neurocytol 20: 844–854. 1783941 10.1007/BF01191735

[59] Kahles F, Findeisen HM, Bruemmer D (2014) Osteopontin: A novel regulator at the cross roads of inflammation, obesity and diabetes. Mol Metab 3: 384–393. doi: 10.1016/j.molmet.2014.03.004 24944898 10.1016/j.molmet.2014.03.004 PMC4060362

[60] Müller D, Cherukuri P, Henningfeld K, Poh CH, Wittler L, Grote P et al (2014) Dlk1 promotes a fast motor neuron biophysical signature required for peak force execution. Science 343: 1264–1266. doi: 10.1126/science.1246448 24626931 10.1126/science.1246448

