# Supplementary figures and images for: Four alpha ganglion cell types in mouse retina: Function, structure, and molecular signatures
Source: PLoS One. 2017 Jul 28;12(7):e0180091. doi: 10.1371/journal.pone.0180091 (PMC5533432; doi:10.1371/journal.pone.0180091)

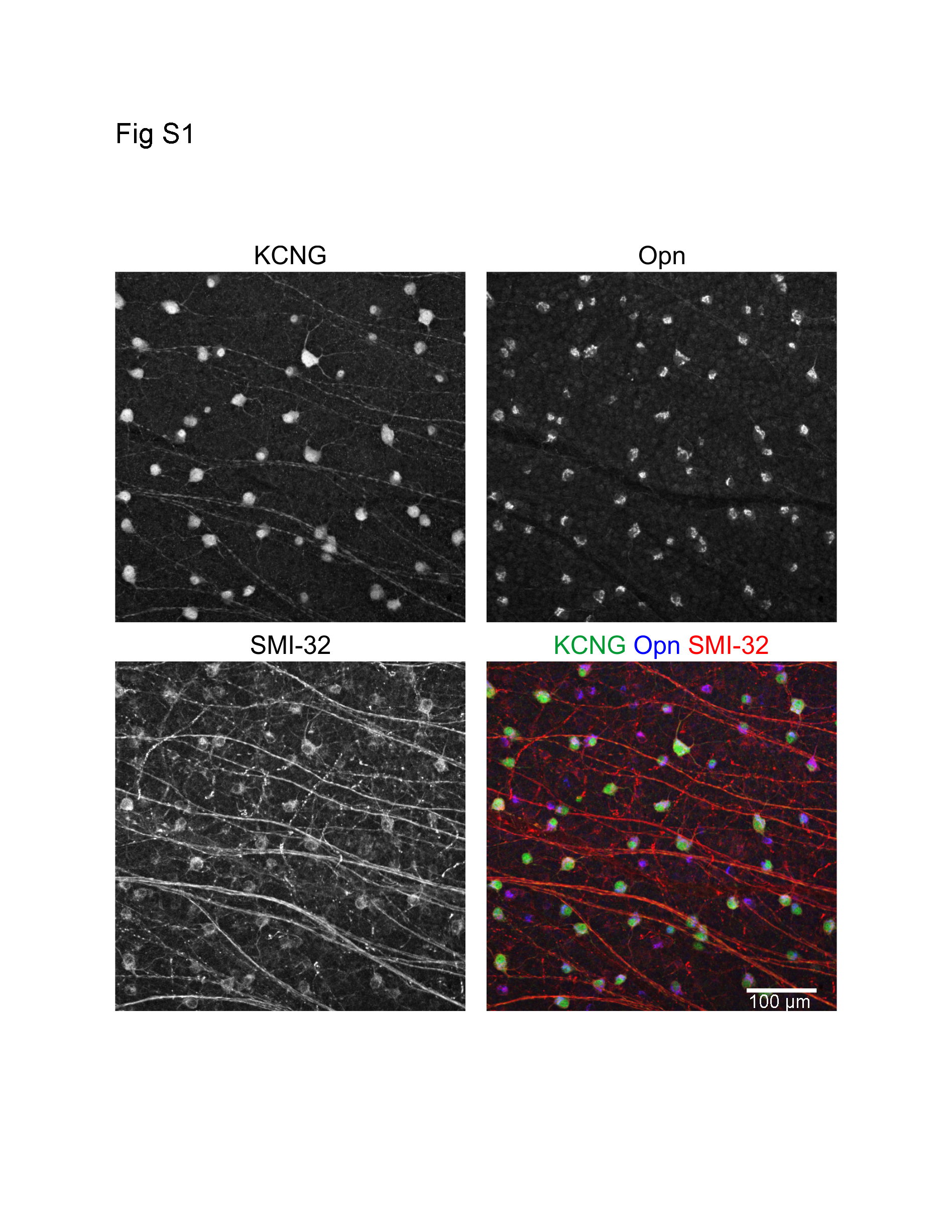

Supplement: S1 Fig — Retina of a KCNG4-cre;thy1-stop-YFP1 mouse stained with antibodies for GFP (KCNG), osteopontin (Opn), and neurofilament (SMI-32), with overlap shown in false color (KCNG Opn SMI-32). Note the strong correspondence among the 3 labels. However, because each of the markers varies somewhat in strength from cell to cell, a binary assignment to “positive” and “negative” necessarily leads to overlap numbers less than 100%, as quoted in the text. (TIFF) [file pone.0180091.s001.tiff]
